# Redesigning the psychiatry induction

**DOI:** 10.1192/bjo.2021.450

**Published:** 2021-06-18

**Authors:** Sharadha Wisidagama, Martin Schmidt

**Affiliations:** Surrey and Borders Partnership NHS Foundation Trust

## Abstract

**Aims:**

To analyse the current psychiatry induction programme with regards to national guidance, local requirements, trainee and trainer feedback and implement recommendations to streamline where possible.

**Background:**

Junior doctors in training rotate every 4 or 6 months depending on the grade/programme group. GP and FY trainees are often new to psychiatry therefore require a comprehensive induction.

Our Trust has had a three day induction for new junior doctors comprised of 1 day Corporate Induction, 1 day Electronic Records Training and 1 day Local induction.

During the 3 day induction programme there is often a service gap with covering out of hours and acute services. Trainees and trainers have expressed concern regarding the service gap.

We therefore embarked on a review of the induction programme to investigate whether it could be improved in content and length of time to deliver.

**Method:**

Review the regulatory bodies requirements for junior doctor induction.

Gain an understanding of the trainees and trainers perspective of the induction programme.

Review the items in the induction programme according to the requirements of the regulatory bodies.

Tailor the induction programme for junior doctors’ needs whilst complying with the regulatory bodies requirements.

**Result:**

The General Medical Council (GMC), British Medical Association (BMA), Gold Guide, Health Education England (HEE) and National Health Service (NHS) employment have no specific statutory and mandatory training requirements for induction.

The regulatory bodies have generic standards for junior doctor induction.

Induction is the responsibility of the Trust.

Trainee perspective: Electronic record system, Mental Health Act (MHA) and pharmacy training were agreed as needing review in terms of its content and length.

Trainees also requested extra items to be included in the induction programme to support successful transition in to their work placements.

The education department met with the Digital Team, MHA Team and Pharmacy Team to develop new and more relevant course content and add in the requested items.

The new induction programme was launched in December 2019 and was reduced in length from 3 to 2 and a half days. Trainee satisfaction improved as evidence by trainee feedback.

**Conclusion:**

The review was helpful in establishing the requirements for a good induction and highlighting areas for improvement.

The new induction was more focussed, shorter in duration and had improved trainee feedback.

The Medical Education Department will assess the changes following the December 2019 induction and continue to review its induction programme.

